# A Rare Case of Delayed Presentation of Acute Midgut Volvulus in an Adolescent

**DOI:** 10.7759/cureus.62256

**Published:** 2024-06-12

**Authors:** Pramod Nichat, Ami S Gandhi, Chetan Rathod, Khadeija Hussain, Nishit Patel

**Affiliations:** 1 General Surgery, Grant Government Medical College and Sir JJ Group of Hospitals, Mumbai, IND; 2 General Surgery, Princess Royal University Hospital, London, GBR

**Keywords:** bilious vomiting, surgical case report, incomplete intestinal rotation, ladd's band, laparoscopic surgery, laparoscopic ladd's procedure, intestinal malrotation, acute midgut volvulus

## Abstract

Intestinal malrotation is primarily diagnosed in the neonatal period, when symptoms typically first appear. In contrast, occurrences in adults are quite uncommon. Adult cases are less frequently reported, often because the condition remains asymptomatic or presents with nonspecific symptoms that can delay diagnosis. Intestinal malrotation in adults can show a range of symptoms, from acute bowel obstruction to vague and chronic symptoms, often leading to a delay in the diagnosis compared to children. Patients with this condition present a distinctive challenge for surgeons regarding diagnosis and treatment, especially in acute situations. This study presents a rare case of a 16-year-old boy who came with chief complaints of acute abdominal pain and multiple episodes of bilious vomiting. The patient underwent an emergency laparoscopy converted to an open Ladd’s procedure. During surgery, the duodenum, small intestine, cecum, and appendix were discovered to be abnormally positioned, and the transverse colon and mesentery were twisted along with the superior mesenteric artery and superior mesenteric vein, with the presence of classical Ladd's bands confirming preoperative CT findings of midgut volvulus. The patient tolerated the procedure well, with no intraoperative complications. Acute midgut volvulus is rare in adults and older children. Still, the differential diagnosis should be kept in mind in patients with pain in the abdomen and multiple episodes of bilious vomiting in cases with an uncertain diagnosis to plan proper management and avoid intraoperative surprises. Early detection, accurate imaging methods, and prompt intervention can mitigate complications that could increase morbidity and mortality.

## Introduction

Intestinal malrotation is most often identified during the neonatal period, with cases in adults being relatively rare, occurring at 0.2% [1]. The circumstances in which the normal embryologic process of intestinal rotation and fixation of the mesentery fails to happen lead to intestinal malrotation.

The Stringer classification has divided malrotation into three types: non-rotation (type I), incomplete rotation (type II) with duodenal malrotation, and reverse rotation (type III) with duodenal plus cecal malrotation [2]. Patients with malrotation have a short root of mesentery, making them more vulnerable to midgut volvulus, a condition that can lead to severe morbidity and mortality [3]. Ensuring an accurate diagnosis followed by prompt surgical intervention is essential to preventing disastrous outcomes [3]. Such patients continue to be a challenge for the surgeon acutely from a diagnostic and treatment point of view. Here, we report a small intestinal malrotation with midgut volvulus in a 16-year-old male patient with type II incomplete malrotation.

## Case presentation

A 16-year-old boy presented with chief complaints of acute onset, colicky pain in the abdomen in the upper right quadrant for six days associated with multiple episodes of bilious vomiting. The pain was relieved by bending forward and aggravated by eating and sleeping. The patient did not have similar complaints in the past and had no previous history of surgery.

On examination, the patient was vitally and hemodynamically stable. Abdomen examination concluded that the abdomen was soft and non-tender, with no guarding or rigidity. No signs of peritonitis were observed. Standard blood tests were within normal limits, encompassing a complete blood count, random blood sugar, serum electrolytes, renal function test, amylase, lipase, liver function test, and clotting profile. The chest radiograph was within normal limits. An abdominal radiograph taken in an erect posture showed a bowel loop filled with gas in the central part of the abdomen. Ultrasonography of the abdomen and pelvis revealed no significant abnormality.

**Figure 1:**
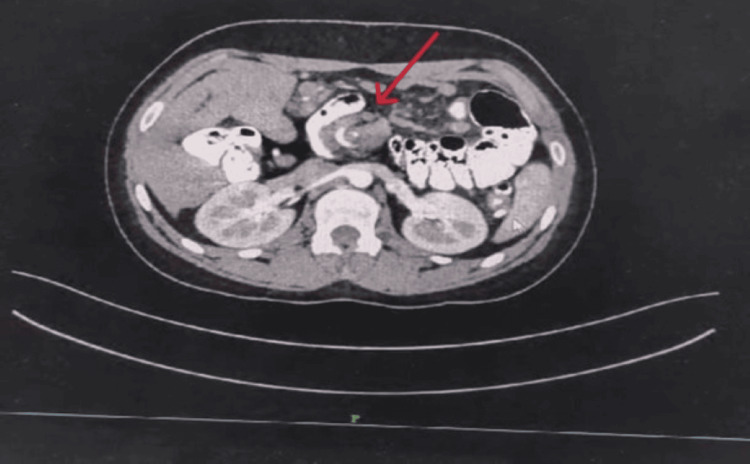
Whirlpool sign in the arterial phase of CECT CECT: contrast-enhanced computed tomography

The diagnosis continued to be unclear until an emergency contrast-enhanced CT (CECT) scan was done with iohexol contrast, which demonstrated features of malrotation. The CECT abdomen and pelvis showed twirling of the mesentery along with the superior mesenteric artery (SMA) and superior mesenteric vein, with twisting of the transverse colon below the duodenojejunal segment without any grossly dilated bowel loops, as seen in Figure 1.

**Figure 2:**
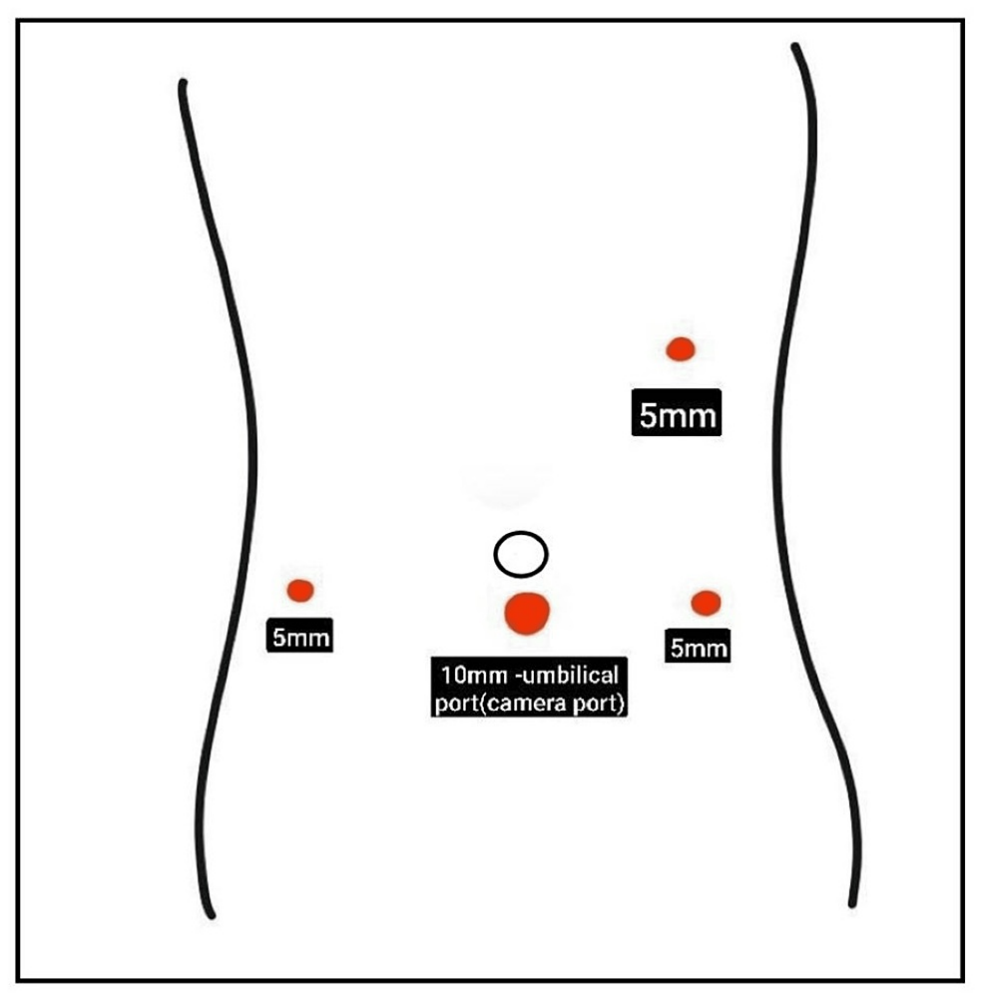
Port placement for the laparoscopy Ladd’s procedure Image Credit: Ami Gandhi

The patient underwent an emergency laparoscopy converted to an open Ladd's procedure under general anesthesia after due consent. Laparoscopic ports were inserted, as shown in Figure 2.

**Figure 3:**
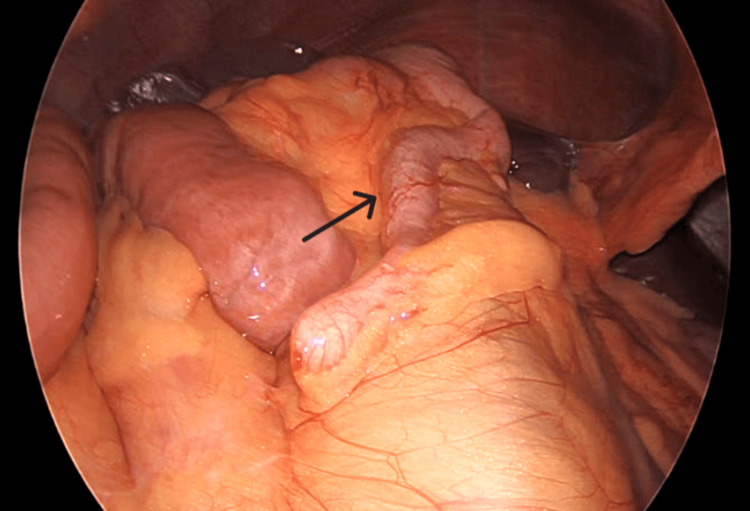
Intraoperative image showing the appendix (black arrow) and ileocecal junction in the subhepatic region

**Figure 4:**
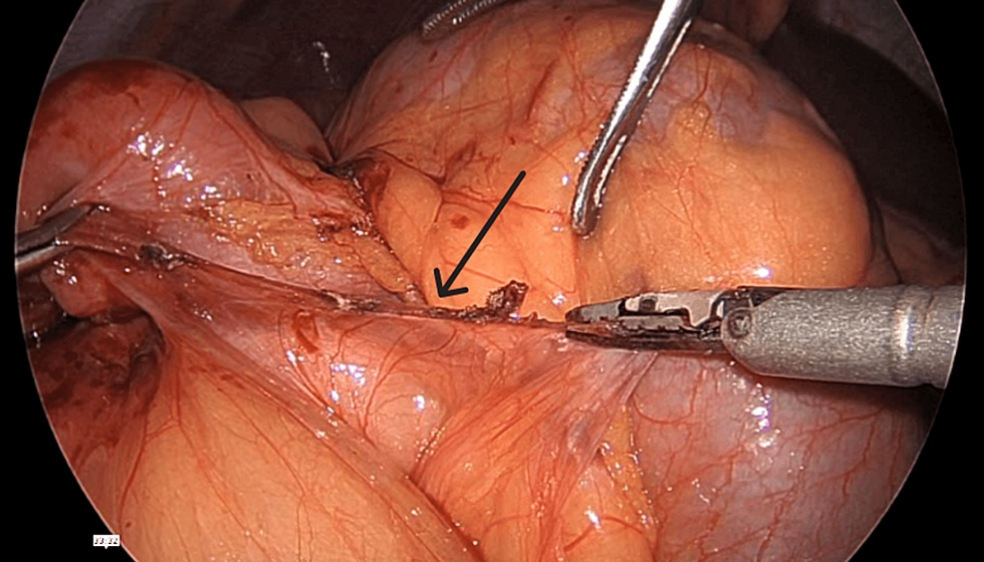
Ladd's band (black arrow) seen adjoining the transverse colon and duodenum

**Figure 5:**
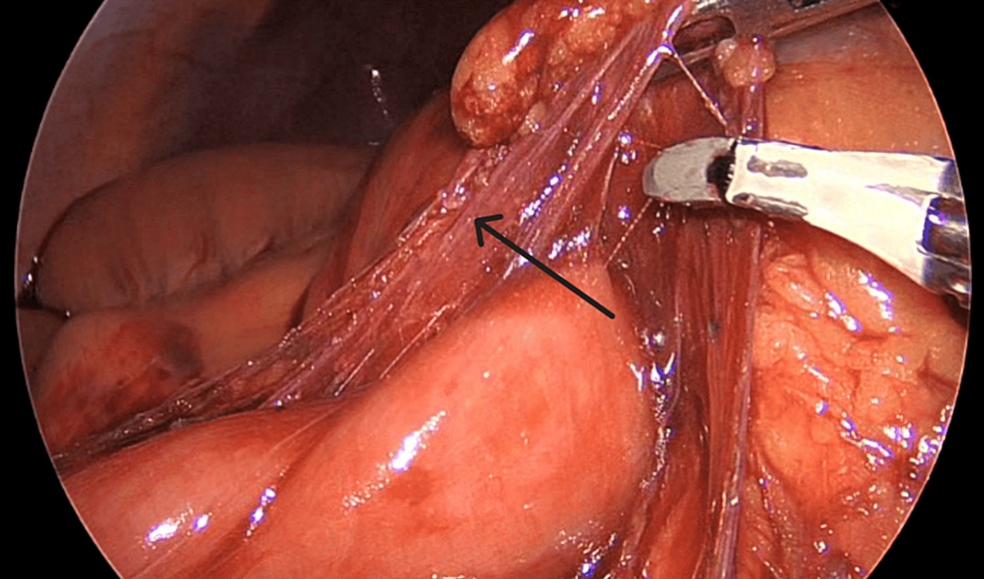
Ladd's band (black arrow) seen near the duodenojejunal segment

**Figure 6:**
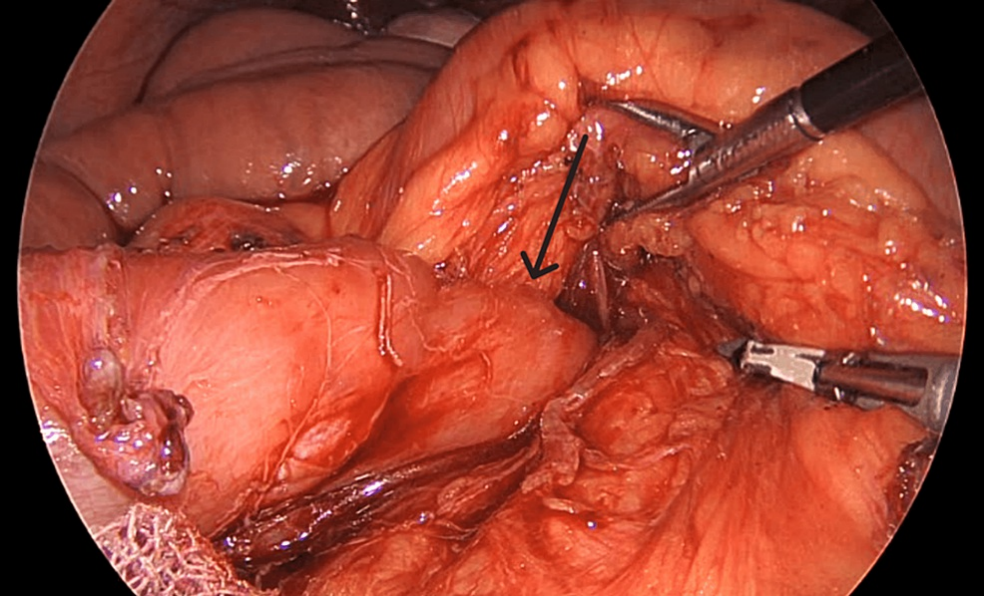
Loop of the transverse colon (black arrow) seen passing underneath the segment of the duodenum

Intraoperatively, during the initial inspection of the abdomen and the insertion of the visual port, an incomplete malrotation of type II was found, as seen in Figure 3. Multiple Ladd’s bands were encountered from the duodenojejunal segment traversing posteriorly to the transverse colon, causing twisting of the colon, as seen in Figure 4 and Figure 5. Further inspection revealed a loop of transverse colon, passing underneath a small intestine, as seen in Figure 6. De-rotation of the transverse colon was tried, and due to technical challenges and difficult ergonomics, the decision to convert the procedure to open was made. A midline abdominal incision was taken, and after reaching the abdominal cavity, de-rotation of the transverse colon was done in an anticlockwise position 180° after Kocherization of the duodenum. The root of the mesentery was broadened, and an appendicectomy was performed. The small intestine on the right side with the cecum and the large intestine on the left side were placed in the abdomen and closed. The patient tolerated the procedure well, with no intraoperative complications.

## Discussion

In its initial stage, the embryonic intestine starts as a straight tube with a common mesentery. Between the fourth and sixth weeks of pregnancy, the midgut grows rapidly and outpaces the slower expansion of the abdominal cavity. As a result, a significant portion of the midgut protrudes into the yolk sac through the umbilical ring, forming a physiological umbilical hernia [4]. From the sixth to the 10th week of embryonic development, the abdominal cavity grows rapidly, increasing its volume. During this time, the midgut returns to the abdominal cavity, rotating 270° counterclockwise around the SMA as its axis [4]. The curve of the proximal small intestine, including the duodenum and jejunum, shifts from right to left, passing behind the SMA to the left, forming the suspensory ligament of the duodenum. The junction of the ileum and colon shifts from left to right in front of the SMA toward the upper right abdomen and then descends to the right iliac region [4]. Once the normal rotation is complete, the transverse colon is positioned anterior to the SMA. Their mesentery anchors the ascending and descending colons to the posterior abdominal wall. The small intestine mesentery attaches obliquely, stretching from the upper left abdomen to the lower right abdomen, including the duodenojejunal segment, and securing the posterior abdominal wall [4]. The duodenojejunal junction is anchored to the SMA at the Treitz ligament, and the cecum is attached to the right side of the lower abdominal wall. The above-mentioned two anchor points of the small bowel mesentery are essential to prevent malrotation of the intestine [4].

Malrotation happens when typical rotation and fixation of the embryonic gut and mesentery don't occur during the 10th-12th week of pregnancy [5]. A narrow base of mesentery and Ladd's bands are formed, predisposing patients to complications like twisting of intestinal loops. Hence, volvulus most commonly presents as an emergency requiring surgery during the first month of life and is less frequent in older children and adults [6]. Intestinal malrotation in adults often exhibits a variable clinical presentation, like acute bowel obstruction, or can have an insidious course with nonspecific symptoms, which often delays diagnosis in comparison to pediatric patients. In older children and adults, the symptoms tend to be more chronic and intermittent, ranging from partial or intermittent duodenal obstruction or a chronic midgut volvulus causing abdominal pain, multiple episodes of bilious vomiting associated with weight loss or failure to thrive, and other non-specific gastrointestinal complaints [6].

The CT findings in midgut volvulus show a classic whirlpool pattern, which is a characteristic feature. It is seen around a central SMA, as shown in Figure 1. CT allows rapid diagnosis of this unusual and rare condition in adult patients [7].

The approach to treating malrotation depends on the severity of the patient's clinical signs and symptoms. Patients with chronic symptoms in the absence of acute volvulus are treated with an elective Ladd's procedure. Emergency laparotomy is necessary for acute intestinal volvulus following appropriate hemodynamic resuscitation [8]. Ladd’s procedure was originally described by pediatric surgeon William Ladd in 1936. It remains the primary approach in surgical management even today. The procedure comprises five steps and has been outlined for open and laparoscopic approaches [9]. The five steps include evaluation for volvulus with de-torsion in a counterclockwise direction, division of Ladd’s band along with the inter-mesenteric division of the band, appendectomy because of its unusual location, and, to prevent confusion in the future, repositioning the bowel in its appropriate anatomical location after broadening the root of mesentery [9].

The laparoscopic method provides superior visualization of the whole abdomen, attachment of the mesentery, and the presence of Ladd’s bands, making it the preferred choice in the majority of cases [10].

## Conclusions

Acute midgut volvulus is infrequent in adults and older children. It is crucial to consider it in the differential diagnosis for patients with acute abdominal symptoms and recurrent episodes of bilious vomiting, particularly in cases where the diagnosis is unclear. This proactive approach ensures appropriate planning for management, reducing the likelihood of unexpected findings during surgery. Early identification through timely diagnosis and precise imaging, followed by prompt intervention, is pivotal in averting potential morbidity and mortality linked with midgut volvulus complications such as intestinal obstruction, bowel ischemia, and intestinal perforation.
